# Mechanism of aromatic amine carcinogen bypass by the Y-family polymerase, Dpo4

**DOI:** 10.1093/nar/gkv1067

**Published:** 2015-10-19

**Authors:** Alfonso Brenlla, David Rueda, Louis J. Romano

**Affiliations:** 1 Department of Chemistry, Wayne State University, Detroit, MI 48202, USA; 2 Department of Medicine, Section of Virology, Imperial College London, London, UK; 3 Single Molecule Imaging Group, MRC Clinical Sciences Centre, Imperial College London, London, UK

## Abstract

Bulky DNA damage inhibits DNA synthesis by replicative polymerases and often requires the action of error prone bypass polymerases. The exact mechanism governing adduct-induced mutagenesis and its dependence on the DNA sequence context remains unclear. In this work, we characterize Dpo4 binding conformations and activity with DNA templates modified with the carcinogenic DNA adducts, 2-aminofluoene (AF) or N-acetyl-2-aminofluorene (AAF), using single-molecule FRET (smFRET) analysis and DNA synthesis extension assays. We find that in the absence of dNTPs, both adducts alter polymerase binding as measured by smFRET, but the addition of dNTPs induces the formation of a ternary complex having what appears to be a conformation similar to the one observed with an unmodified DNA template. We also observe that the misincorporation pathways for each adduct present significant differences: while an AF adduct induces a structure consistent with the previously observed primer-template looped structure, its acetylated counterpart uses a different mechanism, one consistent with a dNTP-stabilized misalignment mechanism.

## INTRODUCTION

The exposure of genetic material to endogenous and exogenous chemical carcinogens can result in the formation of DNA adducts (1–3). DNA damaged in this way can block replication by high-fidelity DNA polymerases leading to double-strand DNA breaks or mutations (4). Blockage of replication at a damaged site can be overcome by the recruitment of Y-family polymerases that allows the bypass of damage in DNA (5,6). It is thought that the wider active site of these polymerases enables them to carry out replication past many forms of bulky adducts in DNA (7). Depending on both the Y-family polymerase and the adduct identity, translesion synthesis (TLS) can occur in an error-free or error-prone manner (8,9) and can result in disease avoidance. For example, a mutation in a gene coding for a Y-family polymerase has been shown to be responsible for Xeroderma pigmentosum variant (XPV) (10,11).An undesired side effect of the ability of Y-family polymerases to bypass DNA damage is a lessened effect of chemotherapeutic agents that exert their effect by damaging DNA. The presence of these polymerases allows tumor cells to avoid DNA-damage induced apoptosis and can result in cancer persistence (4).

**Figure 1.**
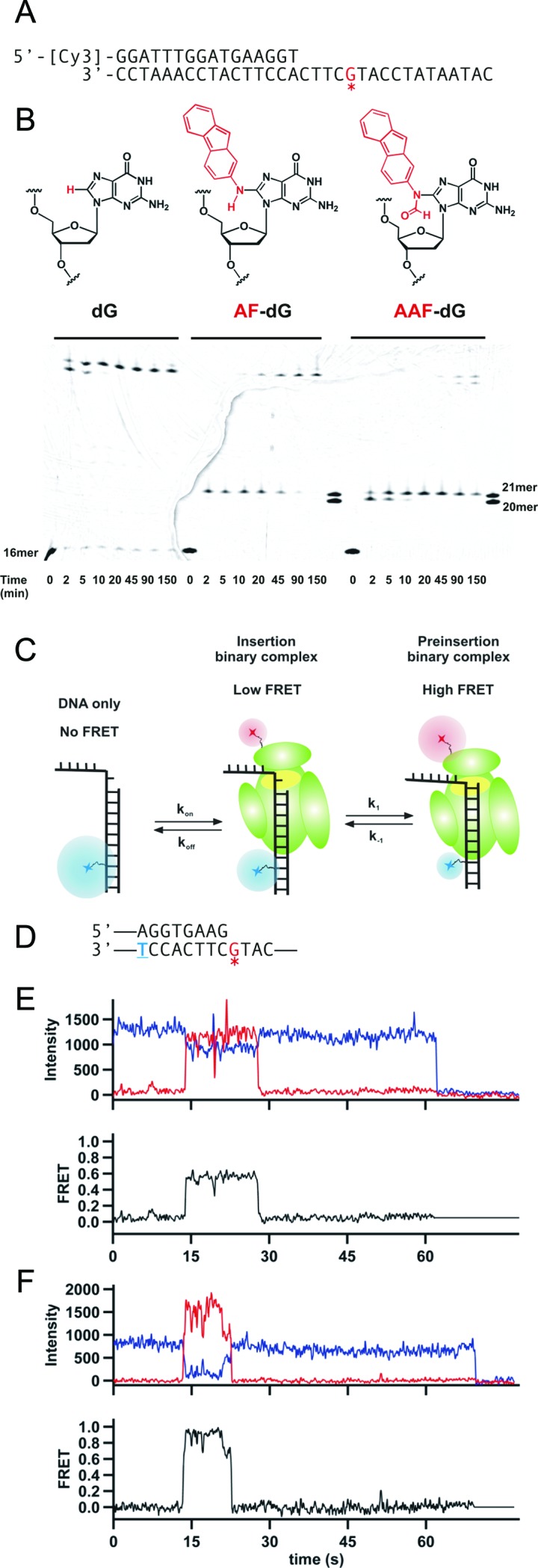
Carcinogenic adducts 2-aminofluoene (AF) and N-acetyl-2-aminoﬂuorene (AAF) induce polymerase stalling at different positions on the DNA. (**A**) Primer-template sequence used to characterize Dpo4 activity around adduct sites. A Cy3 dye for gel imaging is conjugated at the 5′ end of the primer. The asterisk red G in the template corresponds to either an unmodified deoxyguanosine (dG), N-(deoxyguanosin-8-yl)-2-aminoﬂuorene (AF-dG) or N-(deoxyguanosin-8-yl)-N-acetyl-2-aminoﬂuorene (AAF–dG). (**B**) Chemical structure of dG (left), AF–dG (middle) or AAF–dG (right). Below the structures a Running start Dpo4 extension assay is shown. Three different reactions were carried out with the indicated primer-templates. Reactions were initiated by addition of polymerase and quenched at the indicated time points by mixing with equal volume of loading buffer (10 mM EDTA, 1 mg/ml bromophenol blue in formamide). A lane to the right of the reactions with carcinogens marks the position before the adduct (20-mer) and the adduct position (21-mer). (**C**) Schematic of single molecule design and summary of the previously proposed model. Upon polymerase binding, energy is transferred from Cy3 (blue sphere) to the Cy5 on the protein (red sphere). Dpo4 shuttles between two different conformations: an insertion binary complex showing low FRET and a preinsertion binary complex that shows high FRET. (**D**) Primer-template sequence used in the smFRET studies of Dpo4 binding to DNA. A Cy3 dye is conjugated to the underlined blue thymine, while the asterisk red G contains an AF or AAF adduct. (**E**) Characteristic single-molecule trace for Dpo4 binding the AF-modified DNA construct shown in (**D**). Polymerase binding at ∼15 s results in a decrease in Cy3 signal (blue line) accompanied by an increase in Cy5 intensity (red line). The bottom trace shows the FRET efficiency (black) calculated as FRET = IA/(IA + ID). (**F**) Example trace for Dpo4 binding the AAF-modified DNA construct shown in (**D**).

Aromatic amines are a well known type of carcinogens found in a wide variety of sources such as overcooked meats, tobacco smoke or air pollution, making human exposure to these types of carcinogens almost unavoidable (13,14). N-acetyl-2-aminoﬂuorene (AAF) is a model carcinogen (15–17) that yields two different adducts upon reaction at the C8 position of guanine bases, AF–dG and AAF–dG, which differ only by the presence of an acetyl group on the amine linked to the guanine (Figure 1B) (18–20). The presence of this acetyl group in AAF–dG causes significant chemical and biological differences between the two adducts. NMR studies have shown that the AF–dG adduct mostly adopts an *anti* conformation in duplex DNA (21,22), allowing the damaged base to form Watson–Crick hydrogen bonding, while the AAF–dG is found preferentially in a *syn* conformation, with the fluorene ring moiety stacking on the DNA bases and preventing Watson–Crick pairing with the damaged base (23). The characteristic conformations exhibited by each adduct results in different effects on DNA polymerase activity (15,16,24–27). While AF–dG adducts can be bypassed by most replicative DNA polymerases *in vitro*, AAF–dG adducts pose a strong block and can only be bypassed by replicative polymerases in specific sequences, often leading to frameshift mutations (12,28). AAF–dG also acts as a stronger block for Y-family polymerases (29–32), suggesting that the *syn* (AAF–dG) and *anti* (AF–dG) conformations may be somewhat preserved upon polymerase binding. We have shown that Polη/DNA complex is stabilized when the correct dNTP (dCTP) binds across from the AAF–dG (29), suggesting that the *syn* conformation for AAF–dG may be disrupted under these circumstances. The Carell group obtained an interesting crystal structure of Polη with AAF–dG at the active site that may explain the slow bypass observed for this adduct (33). In this structure, AAF–dG maintained a *syn* conformation, but the primer DNA strand was rotated just enough to enable the formation of one hydrogen bond between the templating damaged base and the incoming dNTP.

Dpo4 is a model Y-family polymerase able to bypass a wide variety of DNA lesions (34), including bulky aromatic adducts (35–39). Dpo4 is a homolog to the human Polκ polymerase and this polymerase is thought to be involved in synthesis past AAF adducts in mammalian cells (40). Several studies have found that Dpo4 may use the downstream templating base upon encountering DNA damage (38,41), a trend that is enhanced by the presence of repetitive sequences (36,42,43). These results suggest that Dpo4 may use a dNTP-stabilized or a template slippage mechanism as a function of the DNA sequence.

A Dpo4 crystal structure showed that AF–dG adopts a *syn* conformation when the adduct base is forming an dA:AF–dG mismatch (32). A cognate AF–dG:dC base pair at the active site yields two different complexes, both with the adduct in *anti* conformation. Based on extension assays data, a model was proposed in which AF–dG tends to loop out causing primer template slippage (32). Although it was shown that Dpo4 can bypass AAF–dG (34), no other experimental studies have characterized this process. Computational work by Broyde *et al*. suggests that AAF–dG may exist in both *syn* and *anti* conformations when positioned at the Dpo4 active site (44).

In this work, we used single-molecule FRET and single-nucleotide incorporation assays to characterize the mechanism of bypass of AF–dG and AAF–dG adducts by Dpo4. We find that both AF and AAF–dG adducts inhibit synthesis presumably because the adducts induce a structure that prevents the proper alignment of the template base and the incoming dNTP, as is observed in the Dpo4 crystal structures containing either AF (32) or benzo[a]pyrene adducts (36). However, because these solution studies with Dpo4 and the modified templates show that bypass eventually occurs, it seems likely that the catalytically inactive complex is able to interconvert to an active complex during DNA synthesis. To observe this type of interconversion dynamics in real time, we performed smFRET experiments to monitor the interactions between Dpo4 and templates containing AF or AAF–dG adducts. We find that both adducts distort polymerase binding to DNA, but in the presence of dNTPs, a structure quite similar to the proper ternary complex is observed. Our data also suggest that there are differences in the mutagenic pathways for AF–dG and AAF–dG. While our AF–dG data agree well with the primer-template looped structures previously observed (32), we find AAF–dG appears to induce misincorporations through a different mechanism, one consistent with a dNTP-stabilized misalignment structure where the incoming dNTP is paired with the downstream templating base.

## MATERIALS AND METHODS

### DNA constructs

All DNA oligonucleotides were purchased from Eurofins Operon and purified by HPLC using a reverse-phase C18 column. Templates for single-molecule experiments containing an amino-modified C6-T (Supplementary Table S1) were conjugated to Cy3-NHS-ester (GE Healthcare) using a previously described protocol (45). Oligonucleotides containing Cy3 or biotin at the 5′ position were purchased from Operon and purified by HPLC.

Templates containing AF and AAF adducts were prepared as previously described (46). Briefly, ∼10 nmols of DNA oligonucleotide containing a single guanine were reacted with ∼1 μmol of 2-(N-acetoxy-N-acetyl)aminofluorene (AAAF) by incubating for 2 h at 37°C in 2 mM sodium citrate pH 6.8, 20% ethanol under Argon atmosphere using previously degassed solutions. The reaction was stopped by extracting the excess AAAF with diethyl ether and the AAF-modified template was purified by HPLC as previously described (46). The AAF adduct was converted to an AF adduct by incubating the AAF-modified oligonucleotide in 3 M NaOH and 0.25 M β-mercaptoethanol at 37°C for 45 min as previously described (46). The reaction was quenched by mixing with an equivalent amount of 3 M HCl and the AF-modified template purified by HPLC.

### Dpo4 labeling

*Escherichia coli* RW382 cell line transformed with a plasmid containing the Dpo4 gene was provided by Roger Woodgate (NICHD). Dpo4 was purified to >95% and labeled with Cy5 as previously described (45). The presence of a single Cy5 dye does not affect Dpo4 activity (45).

### Extension assays

Running start extension assays were carried out by incubating 15 nM primer-template (16mer:33mer 1:2 ratio) with 1–100 nM Dpo4 in reaction buffer (50 mM TrisCl pH 7.5, 10 mM MgCl_2_, 100 μM dNTPs, 50 μg/ml BSA and 2 mM DTT) at 30°C for the indicated times. Reactions were started by the addition of polymerase. The same conditions were used for single nucleotide incorporation assays, except reactions were started by the addition of the indicated dNTP and incubated for 20 min. Reactions were quenched by mixing aliquots with equal volumes of 2X loading buffer (10 mM EDTA and 1 mg/ml of bromophenol blue in formamide). Samples were run on 20% denaturing acrylamide gels for ∼16 h at 1000 V and bands visualized by scanning with a Typhoon 9210 phosphoimager (GE Healthcare).

### Single-molecule measurements

DNA duplexes were surface-immobilized via biotin-streptavidin linkage on mPEG-passivated quartz slides as previously shown (45,47). The single-molecule system we used to measure polymerase–DNA interactions was previously described in our prior work (48). Molecules were monitored in buffer containing 50 mM TrisCl pH 7.5, 3.5 mM CaCl_2_, 50 μg/ml BSA and an oxygen scavenging system (protocatechuatedioxygenase from Pseudomonas sp., 5 mM 3,4-dihydroxybenzoic acid and 1 mM Trolox). Fluorescence signal was recorded using a home-built prism-based total internal reflection microscope (49). Measurements were carried out at room temperature (22°C) with 80 ms time resolution. Apparent FRET efﬁciencies were calculated as the acceptor intensity divided by the sum of the donor and acceptor intensities. Time trajectories were analyzed after smoothing with a 3 or 5 point moving average. All FRET trajectories were fit with a 2-state hidden Markov model that allowed the elimination of the 0 FRET peak (see Supplementary Figure S1 for more details). Single-molecule FRET histograms were built from at least 80 individual molecules. The FRET errors were estimated to be ±0.02. All data were analyzed with Matlab. Additional details can be found in Supplementary Information.

## RESULTS

### AF–dG and AAF–dG induce Dpo4 stalling at different positions

It has been previously established that AF and AAF adducts hinder DNA synthesis by Dpo4 (32,34). However, previous publications did not show the exact position on the template that the stalling takes place. We performed a set of running start extension assays using 16mer/33mer primer-template DNA constructs (Figure 1A). Using an unmodified DNA template, Dpo4 was able to carry out full primer extension in less than 2 min, as evidenced by the disappearance of the 16mer primer band and appearance of fully extended product bands (Figure 1B). The two product bands seen after 2 and 5 min of incubation correspond to 33mer and 34mer products, the 34mer resulting from the ability of Dpo4 to incorporate one extra nucleotide in a template-independent manner (50). When an AF-modified template was used as the substrate, Dpo4 transiently paused across from the adduct position (position 21) before bypassing the adduct site and producing the fully extended product. When the AAF-modified template is used, Dpo4 is found to pause at the position one nucleotide before the adduct site (position 20) and then slowly incorporates across from the adduct position. After 150 min incubation, ∼50% of the primers are fully extended and the remainder are stalled at the AAF–dG adduct position. Quantitation of these gel results is shown in Supplementary Table S10–S12. Although it is possible that a decreased binding affinity for modified DNA could explain these results, previous studies have shown that Dpo4 tends to bind damaged DNA with an affinity comparable to unmodified DNA substrates (37,51).

### Effect of adducts located in the templating base position

Our previous smFRET studies have shown that Dpo4 binds to unmodified DNA primer-templates in two different conformations, schematically shown in Figure 1C (45). In this prior study, we presented evidence consistent with crystal structure data (52) that the high FRET state corresponds to a preinsertion complex in which the terminal base pair is located in the active site position and the low FRET state corresponded to the insertion complex that is able to bind an incoming dNTP. We observed transitions between these two states and that only the low FRET state was observed in the presence of the next correct dNTP. When this same smFRET experiment was carried out with templates containing an AF or AAF adduct in the active site (Figure 1D, see Supplementary Table S1 for a list of all the oligonucleotides sequences used), we were able to observe similar traces that showed the interaction between a Cy5-labeled Dpo4 and a Cy3-labeled primer template (Figure 1E and F). As was observed for binding to an unmodified template (45), the donor (Cy3, blue lines) and acceptor (Cy5, red lines) for the traces for both the AF and AAF-modified primer-templates showed anti-correlated fluctuations, indicating Dpo4 binding, ending with a one-step photobleaching event where the Cy3 and Cy5 intensities both drop to zero. The apparent FRET intensities shown in Figure 1E and F, reveal fluctuations between 0 FRET and a higher FRET value that corresponds to the Dpo4 binary complex.

**Figure 2.**
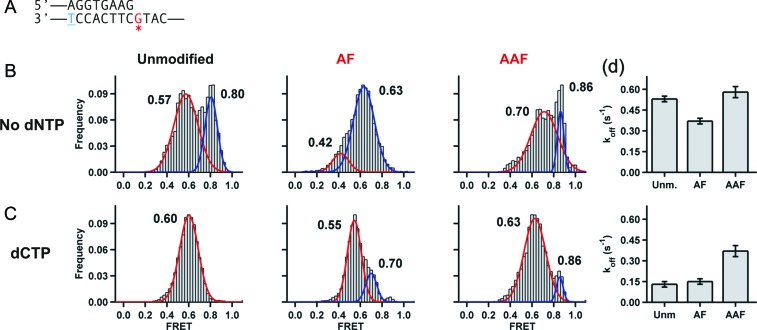
Carcinogenic adducts at the templating base distort polymerase binding to DNA. (**A**) Primer-template sequence used to study Dpo4 binding. A complete sequence is shown in Supplementary Table S1. The blue underlined T in the template is conjugated to a Cy3 dye, while the red starred G marks the position of the carcinogen adducts. (**B**) FRET efficiency histograms for Dpo4 binding to unmodified, AF-modified and AAF-modified primer-templates in the absence of nucleotide. All FRET distributions were fit with two Gaussian functions shown as red and blue lines. (**C**) FRET histograms for Dpo4 binding the same DNA constructs as (**B**) in the presence of the next correct nucleotide dCTP. (**D**) Dpo4 dissociation rates from unmodified, AF-modified and AAF-modified primer-templates for the binary (top) and ternary complexes (bottom).

We next compared the binding modes and dynamics for these templates containing either an AF–dG or AAF–dG adduct at the templating base position (Figure 2A). FRET histograms were calculated by binning at least 80 time trajectories for each construct and, similar to previously published results using a different sequence context, we find that Dpo4 samples two populations when bound to an unmodified template, a low FRET state centered at ∼0.57 and a high FRET state at ∼0.80 (Figure 2B). The quantifications of the Gaussian peaks for all bimodal FRET distributions are provided in Supplementary Information (Supplementary Tables S2–S5). The presence of an AF–dG adduct at the templating position results in a predominant FRET state centered at ∼0.63, with a small overlapping shoulder at ∼0.42. These data show that AF–dG adduct destabilizes the high FRET binary complex and leads to a populating of the lower FRET state (∼0.63). The AAF histogram shows a bimodal FRET distribution with FRET states centered at ∼0.86 and ∼0.70, indicating the formation of significantly altered structures.

As we previously reported for unmodified templates (45), addition of the next correct nucleotide, dCTP in this case, resulted in a single FRET distribution centered at ∼0.60, which, based on crystal structure data, we assigned as the ternary insertion complex (Figure 1C). Similar transitions occur upon the addition of the next correct nucleotide for templates modified with either an AF–dG or AAF–dG adduct, although the positions of the FRET distributions are slightly different and small shoulders having a higher FRET value are observed in each case (Figure 2C). Based on our prior results using unmodified templates (45), it seems likely that the predominant FRET state in each case represents the ternary complex.

We used the dwell time distributions shown in Supplementary Figure S2 to calculate the dissociation rate constants (*k_off_*, Figure 1C) on unmodified and the AF and AAF-modified primer-templates. In agreement with our previous results, a stabilization effect is observed upon dNTP binding with unmodified DNA, as the dissociation rate constant decreases from 0.52 ± 0.02 s^−1^ for the binary complex to 0.13 ± 0.01 s^−1^ for the ternary complex (Figure 2D). A smaller stabilization takes place with AF–dG, which shows a k_off_ = 0.37 ± 0.02 s^−1^ for the binary complex and a k_off_ = 0.15 ± 0.01 s^−1^ for the ternary complex. Interestingly, the presence of an AAF–dG adduct causes the least stabilization when the ternary complex forms. It is possible that the relatively fast dissociation rate (k_off_ = 0.37 ± 0.02 s^−1^) for the ternary complex may contribute to the stalling that occurs across from an AAF–dG adduct.

### Effect of adducts positioned across from the primer terminus

**Figure 3.**
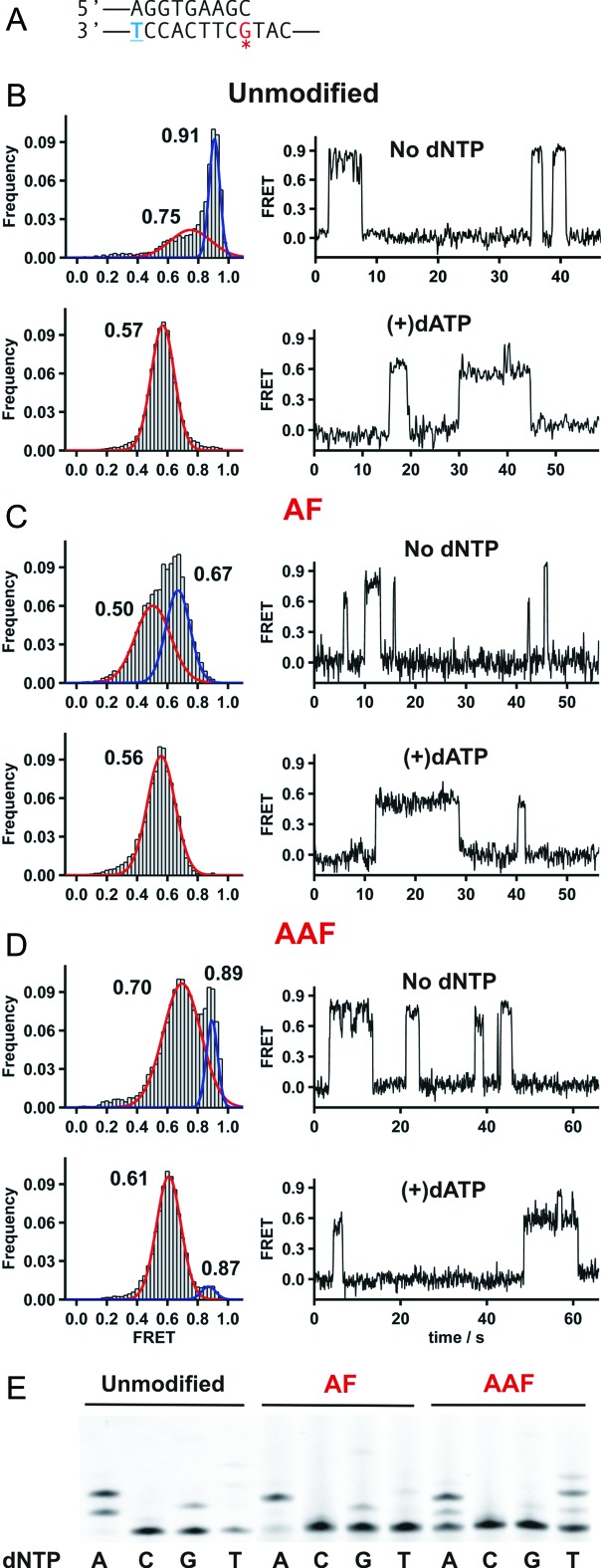
Dpo4 binding to and extending from DNA duplexes with a terminal carcinogenic adduct. (**A**) Primer-template sequence used in this set of experiments. A complete sequence is shown in Supplementary Table S1. The blue underlined T in the template is conjugated to a Cy3 dye, while the red starred G marks the position of the carcinogen adducts. (**B–D**) FRET histograms and representative traces for Dpo4 binding to (**B**) unmodified, (**C**) AF-modified or (**D**) AAF-modified DNA duplex in the absence (top) and presence of the correct nucleotide dATP (bottom). Histograms were fit with Gaussian functions shown here as red or blue lines. (**E**) Single-nucleotide incorporation assays by Dpo4 using unmodified (left), AF-modified (middle) and AAF-modified DNA (right). Reactions were carried out at 37°C in buffer containing 50 mM Tris-HCl, pH 7.5, 10 mM MgCl_2_, 0.025 mg/ml bovine serum albumin, 15 nM primer template (21mer/33mer) and 1/100 nM Dpo4 for unmodified and AF, AAF-modified DNA respectively. Reactions were initiated by the addition of dNTPs (final concentration 100 μM) and incubated for 20 min.

Single-molecule measurements were made using primer-templates in which the primer terminus is positioned across from the AF or AAF-modified G (Figure 3A). As expected (45), for unmodified DNA the FRET distribution shows two peaks at ∼0.75 and ∼0.91 in the absence of dNTP (Figure 3B), while the ternary complex yields a single FRET value of ∼0.57. These values are somewhat higher than expected compared with our previous studies, indicating that the changes in FRET as the primer length is increased can be dependent on the DNA sequence context and the length of the primer-template construct. When AF or AAF–dG adducts were positioned across from the primer terminus, the FRET distributions for the binary complexes showed two peaks (Figure 3C and D), although for the AF case the values were significantly lower (peaks at ∼0.50 and ∼0.67). Dpo4 binding to a terminal AF–dG also showed measurable levels of an alternative type of binding events (Supplementary Figure S3), a phenomenon that will be discussed in future work. In the presence of the next correct nucleotide, (dATP in this case) all three FRET traces (Figure 3B, C and D, bottom) show a major FRET peak at a lower value, each close to ∼0.6, suggesting that in each case a similar ternary complex is formed where the templating base is located in the active site position as part of a ternary insertion complex as was observed with the unmodified template (45). Interestingly, dynamic bursts to a higher FRET state can be observed in the single-molecule traces for the AAF-modified template suggesting that there are alternative conformations that are induced by the presence of the AAF–dG adduct. As a result of these bursts, a small peak centered at 0.87 appears in the FRET histogram.

### Single-nucleotide incorporation from modified primer-templates

To correlate the single-nucleotide FRET states with misincorporation mechanisms, we performed single nucleotide incorporation assays to determine the fidelity of Dpo4 on the unmodified and modified templates. With the unmodified DNA, the correct nucleotide dATP is preferentially incorporated (Figure 3E), although significant levels of dGTP and dTTP are also incorporated. The quantitation of the single nucleotide incorporations are shown in Supplementary Tables S13–S15. Presumably dTTP is incorporated through a misaligned structure as we previously described (45). In the presence of an AF–dG adduct we observe mostly insertion of the next correct nucleotide, dATP and somewhat reduced levels of misincorporations. Interestingly, in the presence of an AAF–dG adduct we observe reduced levels of incorporation and approximately equal levels of dATP and dTTP incorporation. To investigate the different mechanisms that lead to the different incorporation profiles for the AF and AAF modified templates, we next performed single nucleotide extension assays and smFRET experiments with DNA containing terminal mismatches.

### Misincorporation follows a different pathway for AF–dG and AAF–dG adducts

**Figure 4.**
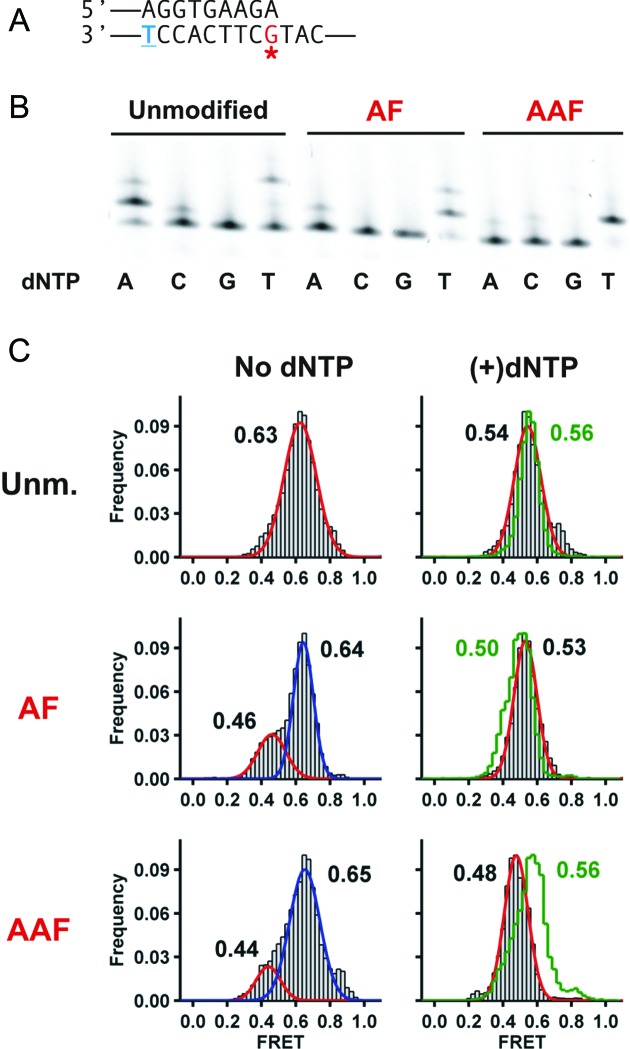
A terminal A:G mismatch distorts Dpo4 binding and increases dTTP misincorporation with AF and AAF-modified DNA. (**A**) Primer-template DNA sequence used in this set of experiments. (**B**) Single-nucleotide incorporation assays by Dpo4 using unmodified (left), AF-modified (middle) and AAF-modified DNA (right). Experimental conditions were the same as in Figure 3E except incubation was carried out at 30°C. (**C**) FRET efficiency histograms for Dpo4 binding to unmodified and AF,AAF-modified DNA in the absence (left) and presence of nucleotides (right). Histograms in the presence of 1 mM dATP are shown as a green line, while 1 mM dTTP are shown as gray bars.

**Figure 5.**
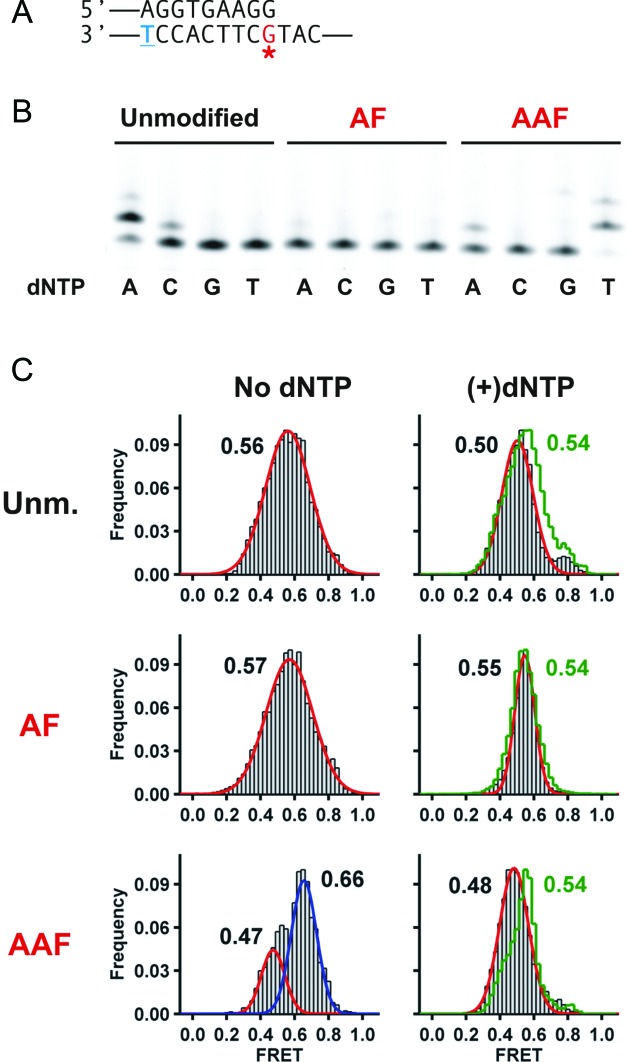
A terminal G:G mismatch distorts Dpo4 binding and constitutes a dead-end for AF DNA but not for AAF DNA. (**A**) Primer-template DNA sequence used in this set of experiments. (**B**) Single-nucleotide incorporation assays by Dpo4 using unmodified (left), AF-modified (middle) and AAF-modified DNA (right). Experimental conditions were the same as in Figure 3E except incubation was carried out at 30°C. (**C**) FRET efficiency histograms for Dpo4 binding to unmodified (Unm), AF or AAF-modified DNA in the absence (left) and presence of nucleotides (right). Histograms in the presence of 1 mM dATP are shown as a green line, while 1 mM dTTP are shown as gray bars.

Prior crystallographic studies (32) showed that the presence of an AF–dG adduct can induce a bulged structure causing the AF–dG to loop out if the terminal nucleotide of the primer can base pair with the nucleotide situated 5′ to the adduct. This structure can lead to the misincorporation of the nucleotide that pairs with the next 5′ nucleotide in the template. We performed a set of experiments using DNA constructs that can and cannot form this type of structure. (Figures 4A and 5A, respectively). According to the model proposed by Rechkoblit *et al*. (32), an AF–dG adduct should loop out in the presence of a terminal A:G mismatch, thus allowing the adjacent dT in the template to pair with the terminal dA from the primer. The data shown in Figure 4B agree with this model and the extension assays with an AF template clearly show preferential incorporation of dTTP over the correct nucleotide dATP. In the absence of an adduct, dTTP is also incorporated but the correct nucleotide, dATP, is preferred, suggesting that primer-template slippage is enhanced in the presence of an AF–dG adduct. The AAF template presents an incorporation pattern with an even greater preference for dTTP misincorporation over dATP.

Single-molecule studies using a DNA duplex with a terminal A:G mismatch in the absence of an adduct shows only one population, with a FRET value of ∼0.63 (Figure 4C). The absence of a second high FRET peak shows that this binary complex cannot form the high FRET complex, which for unmodified templates we assign as the preinsertion complex. In the presence of an AF–dG adduct, the FRET distribution with an A:G mismatch presents two peaks at about the same positions as the observed for a C:G–AF terminal base pair (Figure 3C), showing that the distortion induced by the A:G mismatch in the presence of AF is minimal. The FRET distributions for the AAF–dG case is identical to the AF case, consistent with these structures being similar and implying that the structure for the complex with A:G–AAF is different from the C:G–AAF structure, because the latter structure displays a very different FRET distribution (Figure 3D).

Addition of individual nucleotides dATP or dTTP with the A:G terminal mismatched primer template causes FRET distributions to shift to lower values (Figure 4C), yielding only one population in each case. FRET values are essentially the same for dATP and dTTP with unmodified or AF templates, indicating that binding of either dATP or dTTP results in the formation of complexes with overall similar structures. However, the template containing the A:G–AAF mispair yields a lower FRET value in the presence of dTTP (∼0.48, gray line) when compared to the presence of dATP (∼0.56, green line). These data indicate that with AAF, the structures of the ternary complexes in the presence of dATP versus dTTP are different.

We then determined the effect of having a G:G mismatch at the DNA duplex terminus (Figure 5A). This DNA construct should not be able to loop out the terminal dG because it does not base pair with the dT in the next templating position. Thus, in this case, although the next correct nucleotide, dATP, is incorporated on the unmodified template, it is not surprising that we do not observe incorporation of dTTP on either the unmodified or AF-modified templates (Figure 5B). However, in the case of the template containing an AAF–dG adduct, dTTP is incorporated and is, in fact, the preferred nucleotide, identical to the results observed for the G:A mismatch shown in Figure 4B. This suggests that the misincorporation mechanism for these two mismatched templates containing an AAF–dG adduct is the same. Further evidence for this is the observation that the FRET values for the AAF-modified templates are identical for the G:G mismatch and A:G mismatch in the presence of dTTP (0.48, cf. Figures 4C and 5C).

## DISCUSSION

Many studies have shown that there is a clear link between cancer causation and exposure to exogenous chemical agents (53), and it is also well-established that the majority of these chemical carcinogens can form DNA adducts that can lead to mutations in biological systems (54). It is therefore important to understand the molecular mechanism that leads to a mutation when a DNA polymerase encounters a carcinogenic adduct in DNA. Following treatment of experimental animals with 2-acetylaminofluorene (AAF), two major DNA adducts (AAF–dG and AF–dG) have been observed, each linked to the pathway involved in the metabolic activation process (55). The less distorting AF–dG adduct produces mostly base substitution mutations in bacteria (56), while the more distorting AAF–dG adduct causes mostly frameshifts, often targeted to specific sequences such as GGCGCC (the so-called NarI sequence) (57). Damaged DNA is mostly bypassed by Y-family polymerases *in vivo*, and therefore a characterization of the pathways leading to adduct-induced nucleotide misincorporation is one key to understanding the mechanism by which these adducts result in mutagenesis and cancer.

Previous studies have shown that both AF– and AAF–dG adducts slow the nucleotide incorporation rate by replicative polymerases across from the adduct site, with incorporation across from an AAF–dG adduct occurring extremely slowly (58). We have used single molecule methods to characterize the binding interactions between DNA polymerase I (Klenow fragment) and templates modified with either an AF–dG or AAF–dG adduct and find that neither adduct alters the binary complex when positioned in the templating position but both cause substantial differences when positioned across from the primer terminus. Crystal structures of Dpo4 bound to a primer template having an AF–dG:dC base pair at the active site also showed the formation of distorted structures (32). The single-molecule studies presented here shows that the structures of the Dpo4 complexes with primers ending before or across from either adduct show substantial differences compared to the unmodified structures. We hypothesize that these altered structures are part of the mechanism that allows this polymerase to bypass the AAF–dG adduct, which is a near absolute block for DNA polymerase I.

We also observe that Dpo4 bypasses AAF–dG adduct much more slowly than the AF–dG adduct (Figure 1B). Our single-molecule studies suggests several possible reasons for this slower trans-lesion synthesis. First, unlike what is observed with DNA polymerase I (58,59), Dpo4 shows faster dissociation from templates containing the AAF-dG adduct (Figure 2D), which would lead to reduced levels of synthesis at this position. Second, the binary complex displays FRET states at ∼0.7 and ∼0.86 (Figure 2B), which are considerably different from what is observed for the unmodified complex. It may be that these structures are substantially distorted and therefore unable to readily bind an incoming dNTP. It is interesting that even in the presence of dCTP that the high FRET state at ∼0.86 is retained, suggesting that this structure is unable to bind an incoming nucleotide and form a ternary complex. Surprisingly, we also observe a large population present having a FRET state suggestive of the polymerase binding in a productive ternary complex (∼0.63), although, based on the fact that bypass of the AAF–dG occurs so slowly, we believe that the binding orientation of the nucleotide in this complex may not be optimal for phosphodiester bond formation.

When the primer used is one nucleotide longer, so that the terminus ends across from the adduct position (Figure 3A), the AF and AAF-modified binary complex show FRET states that suggest different structural orientations compared with that observed for the unmodified case (Figure 3B–D). This implies that the adducts are causing substantial distortions in the structure of the complex, resulting in lower FRET states in the case of the AF–dG adduct and very different FRET distributions in the case of the AAF–dG adduct. However, in the presence of the next correct nucleotide (dATP), a single major FRET state is observed for both adducts, suggesting that the nucleotide has bound to the polymerase and caused the formation of a productive ternary complex a process similar to what we observe for the unmodified template (45). Gel analysis confirm this conclusion since dATP is incorporated in each case, although considerably more slowly with the AAF–dG adduct (Figure 3E). This gel analysis shows that dTTP is also incorporated past the AAF–dG adduct but that only dATP is incorporated for the AF–dG adduct.

**Figure 6.**
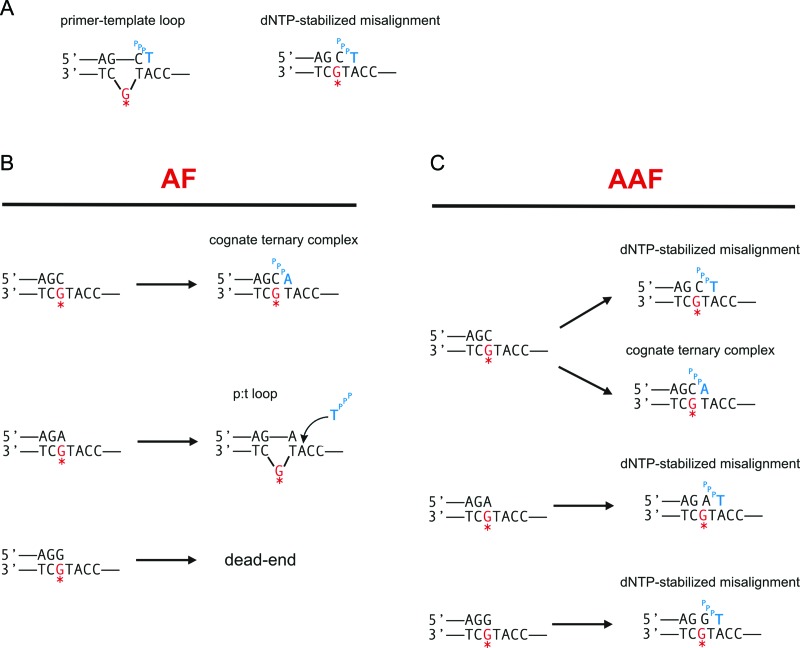
Proposed model for Dpo4 bypassing adducts at the terminal DNA duplex. (**A**) Misaligned DNA structures that allow dTTP misincorporation. In a primer-template loop (left), the terminal primer base, dC, pairs with the downstream T, causing the adducted dG in the template to loop out. In a dNTP-stabilized misaligned structure (right), the incoming dTTP base pairs with the downstream A without looping out; (**B**) In the presence of AF, correct dATP incorporation is preferred with a terminal cognate base pair but in the presence of an A:G mismatch a primer-template slippage occurs, leading to dTTP misincorporation. A terminal G:G mismatch constitutes a dead end for AF. (**C**) In the presence of AAF, a dNTP-stabilized misalignment is preferred, leading to an increased dTTP misincorporation in all cases.

We have considered two potential mechanisms that might account for the incorporation of dTTP at the position 5′ to the AAF–dG adduct, each capable of occurring because of the relatively spacious active site present in this polymerase (32). In the first, a loop could form at the adduct position with the terminal base in the primer pairing with the n+1 position 5′ to the adduct (Figure 6A). Alternatively, a dNTP-stabilized misalignment could occur similar to what we observed for unmodified DNA (45), in which the primer is extended further along the template in the presence of the nucleotide that pairs with the position 5′ to the adduct (Figure 6A). The latter mechanism should lead to a lower FRET state because the polymerase would be positioned about one nucleotide further along the template. The former mechanism should provide similar FRET states as observed for an structure not containing a loop. Using a primer where the terminal nucleotide can pair with the n+1 position we observed identical FRET states in the presence of either dATP or dTTP for the AF–dG (Figure 4B) suggesting that incorporation of dTTP occurs through a looped structure. However, for the AAF–dG case, the presence of dTTP resulted in a lower FRET state, evidence that a dNTP stabilized misaligned structure may have formed. This conclusion is strengthened by the fact that a G:G–AAF mispaired template (Figure 5), which cannot pair with the n+1 position, also efficiently incorporated dTTP and also gives a lower FRET state in the presence of dTTP (Figure 5B).

Taken together, these results suggest that the mechanisms by which misincorporations at the n+1 position 5′ to the adduct are different for an AF and AAF–dG adduct. For the AF case, misincorporation occurs only if the nucleotide across from the adduct can pair with the base at the n+1 position suggesting that a looped out structure had formed by allowing n+2 nucleotide to serve as the template for incorporation (p:t loop in Figure 6B). For the AAF case, misincorporation does not appear to require pairing between the nucleotide across from the adduct with the base at the n+1 position. The FRET states in this case are consistent with the formation of a dNTP-stabilized misaligned structure (Figure 6C).

## Supplementary Material

SUPPLEMENTARY DATA

## References

[1] Poirier M.C. (2004). Chemical-induced DNA damage and human cancer risk. Nat. Rev. Cancer.

[2] Hendriks G., van de Water B., Schoonen W., Vrieling H. (2013). Cellular-signaling pathways unveil the carcinogenic potential of chemicals. J. Appl. Toxicol..

[3] Preston R.J. (2013). DNA reactivity as a mode of action and its relevance to cancer risk assessment. Toxicol. Pathol..

[4] Lange S.S., Takata K., Wood R.D. (2011). DNA polymerases and cancer. Nat. Rev. Cancer.

[5] Guo C., Kosarek-Stancel J.N., Tang T.S., Friedberg E.C. (2009). Y-family DNA polymerases in mammalian cells. Cell. Mol. Life Sci..

[6] Sharma S., Helchowski C.M., Canman C.E. (2013). The roles of DNA polymerase zeta and the Y family DNA polymerases in promoting or preventing genome instability. Mutat. Res..

[7] Yang W., Woodgate R. (2007). What a difference a decade makes: insights into translesion DNA synthesis. Proc. Natl. Acad. Sci. U.S.A..

[8] Yang W. (2003). Damage repair DNA polymerases Y. Curr. Opin. Struct. Biol..

[9] Sale J.E., Lehmann A.R., Woodgate R. (2012). Y-family DNA polymerases and their role in tolerance of cellular DNA damage. Nat. Rev. Mol. Cell Biol..

[10] Masutani C., Kusumoto R., Yamada A., Dohmae N., Yokoi M., Yuasa M., Araki M., Iwai S., Takio K., Hanaoka F. (1999). The XPV (xeroderma pigmentosum variant) gene encodes human DNA polymerase eta. Nature.

[11] Johnson R.E., Kondratick C.M., Prakash S., Prakash L. (1999). hRAD30 mutations in the variant form of xeroderma pigmentosum. Science (New York, N.Y.).

[12] Fuchs R.P., Schwartz N., Daune M.P. (1981). Hot spots of frameshift mutations induced by the ultimate carcinogen N-acetoxy-N-2-acetylaminofluorene. Nature.

[13] Vineis P. (1994). Epidemiology of cancer from exposure to arylamines. Environ. Health Perspect..

[14] Yu M.C., Skipper P.L., Tannenbaum S.R., Chan K.K., Ross R.K. (2002). Arylamine exposures and bladder cancer risk. Mutat. Res..

[15] Shibutani S., Suzuki N., Grollman A.P. (1998). Mutagenic specificity of (acetylamino)fluorene-derived DNA adducts in mammalian cells. Biochemistry.

[16] Tebbs R.S., Romano L.J. (1994). Mutagenesis at a site-specifically modified NarI sequence by acetylated and deacetylated aminofluorene adducts. Biochemistry.

[17] Vu V.T., Moller M.E., Grantham P.H., Wirth P.J., Thorgeirsson S.S. (1985). Association between DNA strand breaks and specific DNA adducts in murine hepatocytes following in vivo and in vitro exposure to N-hydroxy-2-acetylaminofluorene and N-acetoxy-2-acetylaminofluorene. Carcinogenesis.

[18] Kim D., Guengerich F.P. (2005). Cytochrome P450 activation of arylamines and heterocyclic amines. Annu. Rev. Pharmacol. Toxicol..

[19] Kriek E., Miller J.A., Juhl U., Miller E.C. (1967). 8-(N-2-fluorenylacetamido)guanosine, an arylamidation reaction product of guanosine and the carcinogen N-acetoxy-N-2-fluorenylacetamide in neutral solution. Biochemistry.

[20] Beland F.A., Kadlubar F.F. (1985). Formation and persistence of arylamine DNA adducts in vivo. Environ. Health Perspect..

[21] Patel D.J., Mao B., Gu Z., Hingerty B.E., Gorin A., Basu A.K., Broyde S. (1998). Nuclear magnetic resonance solution structures of covalent aromatic amine-DNA adducts and their mutagenic relevance. Chem. Res. Toxicol..

[22] Eckel L.M., Krugh T.R. (1994). 2-Aminofluorene modified DNA duplex exists in two interchangeable conformations. Nat. Struct. Biol..

[23] O'Handley S.F., Sanford D.G., Xu R., Lester C.C., Hingerty B.E., Broyde S., Krugh T.R. (1993). Structural characterization of an N-acetyl-2-aminofluorene (AAF) modified DNA oligomer by NMR, energy minimization, and molecular dynamics. Biochemistry.

[24] Tan X., Suzuki N., Grollman A.P., Shibutani S. (2002). Mutagenic events in Escherichia coli and mammalian cells generated in response to acetylaminofluorene-derived DNA adducts positioned in the Nar I restriction enzyme site. Biochemistry.

[25] Reid T.M., Lee M.S., King C.M. (1990). Mutagenesis by site-specific arylamine adducts in plasmid DNA: enhancing replication of the adducted strand alters mutation frequency. Biochemistry.

[26] Gupta P.K., Johnson D.L., Reid T.M., Lee M.S., Romano L.J., King C.M. (1989). Mutagenesis by single site-specific arylamine-DNA adducts. Induction of mutations at multiple sites. J. Biol. Chem..

[27] Mah M.C., Maher V.M., Thomas H., Reid T.M., King C.M., McCormick J.J. (1989). Mutations induced by aminofluorene-DNA adducts during replication in human cells. Carcinogenesis.

[28] Lambert I.B., Napolitano R.L., Fuchs R.P. (1992). Carcinogen-induced frameshift mutagenesis in repetitive sequences. Proc. Natl. Acad. Sci. U.S.A..

[29] Vooradi V., Romano L.J. (2009). Effect of N-2-acetylaminofluorene and 2-aminofluorene adducts on DNA binding and synthesis by yeast DNA polymerase eta. Biochemistry.

[30] Yuan F., Zhang Y., Rajpal D.K., Wu X., Guo D., Wang M., Taylor J.S., Wang Z. (2000). Specificity of DNA lesion bypass by the yeast DNA polymerase eta. J. Biol. Chem..

[31] Masutani C., Kusumoto R., Iwai S., Hanaoka F. (2000). Mechanisms of accurate translesion synthesis by human DNA polymerase eta. EMBO J..

[32] Rechkoblit O., Kolbanovskiy A., Malinina L., Geacintov N.E., Broyde S., Patel D.J. (2010). Mechanism of error-free and semitargeted mutagenic bypass of an aromatic amine lesion by Y-family polymerase Dpo4. Nat. Struct. Mol. Biol..

[33] Schorr S., Schneider S., Lammens K., Hopfner K.P., Carell T. (2010). Mechanism of replication blocking and bypass of Y-family polymerase {eta} by bulky acetylaminofluorene DNA adducts. Proc. Natl. Acad. Sci. U.S.A..

[34] Boudsocq F., Iwai S., Hanaoka F., Woodgate R. (2001). Sulfolobus solfataricus P2 DNA polymerase IV (Dpo4): an archaeal DinB-like DNA polymerase with lesion-bypass properties akin to eukaryotic poleta. Nucleic Acids Res..

[35] Kirouac K.N., Basu A.K., Ling H. (2013). Structural mechanism of replication stalling on a bulky amino-polycyclic aromatic hydrocarbon DNA adduct by a y family DNA polymerase. J. Mol. Biol..

[36] Ling H., Sayer J.M., Plosky B.S., Yagi H., Boudsocq F., Woodgate R., Jerina D.M., Yang W. (2004). Crystal structure of a benzo[a]pyrene diol epoxide adduct in a ternary complex with a DNA polymerase. Proc. Nat. Acad. Sci. U.S.A..

[37] Sherrer S.M., Brown J.A., Pack L.R., Jasti V.P., Fowler J.D., Basu A.K., Suo Z. (2009). Mechanistic studies of the bypass of a bulky single-base lesion catalyzed by a Y-family DNA polymerase. J. Biol. Chem..

[38] Bauer J., Xing G., Yagi H., Sayer J.M., Jerina D.M., Ling H. (2007). A structural gap in Dpo4 supports mutagenic bypass of a major benzo[a]pyrene dG adduct in DNA through template misalignment. Proc. Natl. Acad. Sci. U.S.A..

[39] Zang H., Chowdhury G., Angel K.C., Harris T.M., Guengerich F.P. (2006). Translesion synthesis across polycyclic aromatic hydrocarbon diol epoxide adducts of deoxyadenosine by Sulfolobus solfataricus DNA polymerase Dpo4. Chem. Res. Toxicol..

[40] Suzuki N., Ohashi E., Hayashi K., Ohmori H., Grollman A.P., Shibutani S. (2001). Translesional synthesis past acetylaminofluorene-derived DNA adducts catalyzed by human DNA polymerase kappa and Escherichia coli DNA polymerase IV. Biochemistry.

[41] Zang H., Goodenough A.K., Choi J.Y., Irimia A., Loukachevitch L.V., Kozekov I.D., Angel K.C., Rizzo C.J., Egli M., Guengerich F.P. (2005). DNA adduct bypass polymerization by Sulfolobus solfataricus DNA polymerase Dpo4: analysis and crystal structures of multiple base pair substitution and frameshift products with the adduct 1,N2-ethenoguanine. J. Biol. Chem..

[42] Xu P., Oum L., Geacintov N.E., Broyde S. (2008). Nucleotide selectivity opposite a benzo[a]pyrene-derived N2-dG adduct in a Y-family DNA polymerase: a 5′-slippage mechanism. Biochemistry.

[43] Wu Y., Wilson R.C., Pata J.D. (2011). The Y-family DNA polymerase Dpo4 uses a template slippage mechanism to create single-base deletions. J. Bacteriol..

[44] Wang L., Broyde S. (2006). A new anti conformation for N-(deoxyguanosin-8-yl)-2-acetylaminofluorene (AAF-dG) allows Watson-Crick pairing in the Sulfolobus solfataricus P2 DNA polymerase IV (Dpo4). Nucleic Acids Res..

[45] Brenlla A., Markiewicz R.P., Rueda D., Romano L.J. (2014). Nucleotide selection by the Y-family DNA polymerase Dpo4 involves template translocation and misalignment. Nucleic Acids Res..

[46] Dutta S., Li Y., Johnson D., Dzantiev L., Richardson C.C., Romano L.J., Ellenberger T. (2004). Crystal structures of 2-acetylaminofluorene and 2-aminofluorene in complex with T7 DNA polymerase reveal mechanisms of mutagenesis. Proc. Natl. Acad. Sci. U.S.A..

[47] Lamichhane R., Solem A., Black W., Rueda D. (2010). Single-molecule FRET of protein-nucleic acid and protein-protein complexes: surface passivation and immobilization. Methods (San Diego, Calif.).

[48] Markiewicz R.P., Vrtis K.B., Rueda D., Romano L.J. (2012). Single-molecule microscopy reveals new insights into nucleotide selection by DNA polymerase I. Nucleic Acids Res.

[49] Zhao R., Rueda D. (2009). RNA folding dynamics by single-molecule fluorescence resonance energy transfer. Methods (San Diego, Calif.).

[50] Fiala K.A., Brown J.A., Ling H., Kshetry A.K., Zhang J., Taylor J.S., Yang W., Suo Z. (2007). Mechanism of template-independent nucleotide incorporation catalyzed by a template-dependent DNA polymerase. J. Mol. Biol..

[51] Lim S., Song I., Guengerich F.P., Choi J.Y. (2012). Effects of N(2)-alkylguanine, O(6)-alkylguanine, and abasic lesions on DNA binding and bypass synthesis by the euryarchaeal B-family DNA polymerase vent (exo(-)). Chem. Res. Toxicol..

[52] Rechkoblit O., Malinina L., Cheng Y., Kuryavyi V., Broyde S., Geacintov N.E., Patel D.J. (2006). Stepwise translocation of Dpo4 polymerase during error-free bypass of an oxoG lesion. PLoS Biol..

[53] Poirier M.C. (2012). Chemical-induced DNA damage and human cancer risk. Discov. Med..

[54] Wogan G.N., Hecht S.S., Felton J.S., Conney A.H., Loeb L.A. (2004). Environmental and chemical carcinogenesis. Semin. Cancer Biol..

[55] Kriek E. (1992). Fifty years of research on N-acetyl-2-aminofluorene, one of the most versatile compounds in experimental cancer research. J. Cancer Res. Clin. Oncol..

[56] Koffel-Schwartz N., Verdier J.M., Bichara M., Freund A.M., Daune M.P., Fuchs R.P. (1984). Carcinogen-induced mutation spectrum in wild-type, uvrA and umuC strains of Escherichia coli. Strain specificity and mutation-prone sequences. J. Mol. Biol..

[57] Hoffmann G.R., Fuchs R.P. (1997). Mechanisms of frameshift mutations: insight from aromatic amines. Chem. Res. Toxicol..

[58] Dzantiev L., Romano L.J. (1999). Interaction of Escherichia coli DNA polymerase I (Klenow fragment) with primer-templates containing N-acetyl-2-aminofluorene or N-2-aminofluorene adducts in the active site. J. Biol. Chem..

[59] Vrtis K.B., Markiewicz R.P., Romano L.J., Rueda D. (2013). Carcinogenic adducts induce distinct DNA polymerase binding orientations. Nucleic Acids Res..

