# Supplementary material for: Mechanism of aromatic amine carcinogen bypass by the Y-family polymerase, Dpo4
Source: Nucleic Acids Res. 2015 Oct 19;43(20):9918–27. doi: 10.1093/nar/gkv1067 (PMC4787768; doi:10.1093/nar/gkv1067)
Supplement: SUPPLEMENTARY DATA [file supp_43_20_9918__index.html]

Mechanism of aromatic amine carcinogen bypass by the Y-family polymerase, Dpo4 — SUPPLEMENTARY DATA 

# Mechanism of aromatic amine carcinogen bypass by the Y-family polymerase, Dpo4

## SUPPLEMENTARY DATA

- SUPPLEMENTARY DATA
