# Supplementary material for: Mechanism of aromatic amine carcinogen bypass by the Y-family polymerase, Dpo4
Source: Nucleic Acids Res. 2015 Oct 19;43(20):9918–27. doi: 10.1093/nar/gkv1067 (PMC4787768; doi:10.1093/nar/gkv1067)
Supplement: SUPPLEMENTARY DATA [file supp_gkv1067_nar-01640-d-2015-File008.docx]

**Supplementary information**

**Supplementary Table S1**. DNA sequences for ensemble and single-molecule experiments^1,2^.

| Extension gel assay oligonucleotides | |
| --- | --- |
| 16mer-Cy3 primer | 5’-[Cy3]-GGA TTT GGA TGA AGG T-3’ |
| 21mer-Cy3 primer | 5’-[Cy3]-GGA TTT GGA TGA AGG TGA AGC-3’ |
| 21mer-Cy3 single A mm primer | 5’-[Cy3]-GGA TTT GGA TGA AGG TGA AGA-3’ |
| 21mer-Cy3 single G mm primer | 5’-[Cy3]-GGA TTT GGA TGA AGG TGA AGG-3’ |
| 33mer template | 3’-CCT AAA CCT ACT TCC ACT TC**G** TAC CTA TAA TAC-5’ |
| Single molecule oligonucleotides | |
| 20mer-biotin primer | 5’-[Biotin]-GGA TTT GGA TGA AGG TGA AG-3’ |
| 21mer-biotin primer | 5’-[Biotin]-GGA TTT GGA TGA AGG TGA AGC-3’ |
| 21mer-biotin single A mm primer | 5’-[Biotin]-GGA TTT GGA TGA AGG TGA AGA-3’ |
| 21mer-biotin single G mm primer | 5’-[Biotin]-GGA TTT GGA TGA AGG TGA AGG-3’ |
| 33mer-Cy3 template | 3’-CCT AAA CCT ACT **T**CC ACT TC**G** TAC CTA TAA TAC-5’ |

^1^ Underlined red G in the templates was either unmodified or modified with AF or AAF adducts.

^2^ Underlined blue T in the single-molecule template was conjugated to Cy3.

**Single-molecule experiments preparation**

Single-molecule slides were prepared as previously described ([1](#_ENREF_1),[2](#_ENREF_2)). DNA was surface-immobilized via biotin–streptavidin–biotin linkage as follows. The slide was incubated 10 min with 0.02 mg/ml streptavidin, washed and incubated another 10 min with 50 pM biotinylated primer-template duplex. After a final washing step, we typically observed 200-300 immobilized DNA molecules per field of view. Dpo4 was introduced in solution (10 nM) with 50 mM Tris-HCl, pH 7.5, 3.5 mM CaCl_2_, 50 mg/ml bovine serum albumin (BSA) and 0/1 mM dNTP in the presence of an oxygen scavenging system/antiblinking cocktail (protocatechuate dioxygenase from Pseudomonas sp., 5 mM 3,4-dihydroxybenzoic acid and 1 mM Trolox).

**Single-molecule FRET setup**

Fluorescence intensity was monitored using a home-built prism-based total internal reflection microscope setup previously described ([3](#_ENREF_3)). Briefly, a green laser beam (532 nm, 25 mW, CrystaLaser CL532-025 -S, Reno, NV) reaches the slide trough a quartz Pellin-Broca prism (CVI Melles-Griot, Albuquerque, NM) at an incident angle larger than the critical angle, creating an evanescence wave that selectively excites the immobilized DNA molecules. Incident laser power is 2 mW. Fluorescence is collected with a single-molecule objective (60x, water immersion, Numerical Aperture = 1.2, Olympus, Center Valley, PA) placed on an inverted microscope (IX-71, Olympus, Center Valley, PA). Scattered laser light is eliminated by a long pass filter (E550LP, Chroma, Rockingham, VT) and Cy3/Cy5 signals are splitted into 2 separate images by a dichroic mirror (DM1,635DCXR, Chroma, Rockingham, VT). These two images reach different halves of a CCD camera (Ixon+, DV-897E, Andor, South Windsor, CT) continuously recording 80 ms time frames.

**Hidden Markov model (HMM)**

Underlying discrete FRET states can often be obscured by the noise inherent to smFRET trajectories. In these circumstances, Hidden Markov modeling (HMM) becomes a very useful technique as it enables reconstruction of an idealized FRET trace from noisy data. HMM assumes that 1) the transitions between different FRET states are governed by single-exponential kinetics and 2) the widening of the FRET distribution due to noise can be modeled by a Gaussian function. Under these 2 assumptions, HMM finds the most likely sequence of states for a given FRET trajectory. A more detailed explanation of HMM applied to smFRET trajectories analysis can be found in Ref. ([4](#_ENREF_4)).

**Analysis of smFRET data**

Supplementary Fig. S1 shows a typical smFRET trajectory. Dpo4 binding events at ~20, 85 and 100 seconds result in an anticorrelated decrease in Cy3 signal (blue) and increased Cy5 signal (red, see top trace). Apparent FRET values are calculated as FRET = I_A_/(I_A_+I_D_) and shown on the bottom trace (black line). The whole range of FRET values is binned in 0.025 width intervals and the number of FRET points (bottom trace in Fig. S1a) that fall within each of those 0.025 intervals is counted to yield the bimodal histogram show in Fig. S1b. The peak centered at FRET 0 corresponds to DNA only, while the peak at FRET ~0.7 represents Dpo4 bound to DNA. Since only the high FRET peak yields useful information, we excluded the 0 FRET points for further analysis in this work. A 2-state hidden Markov model fit of the FRET trajectory (red line, see bottom trace) enables us to index 0 and high FRET data points, allowing us to construct FRET histograms excluding 0 FRET points (Fig S1c). The FRET histograms shown in this work were typically constructed by combining >80 single-molecule trajectories.

Dwell-time distributions were built from the time Dpo4 is bound to DNA (t_on_ in Fig. S1a). For example, the trajectory in Fig. S1a shows three binding vents. The first one lasts 25 seconds (Dpo4 binds to DNA at t = 19 seconds and dissociates at t = 44 seconds), while the second and third binding events are shorter lasting ~2 and 5 seconds respectively. To construct dwell-time histograms (Fig. S3), we typically divided a 0-30 seconds time range in 200 ms time bins and count the number of binding events that fall within a specific time interval. The distribution is then fitted to a monoexponential decay function that yields the estimated dissociation rates.


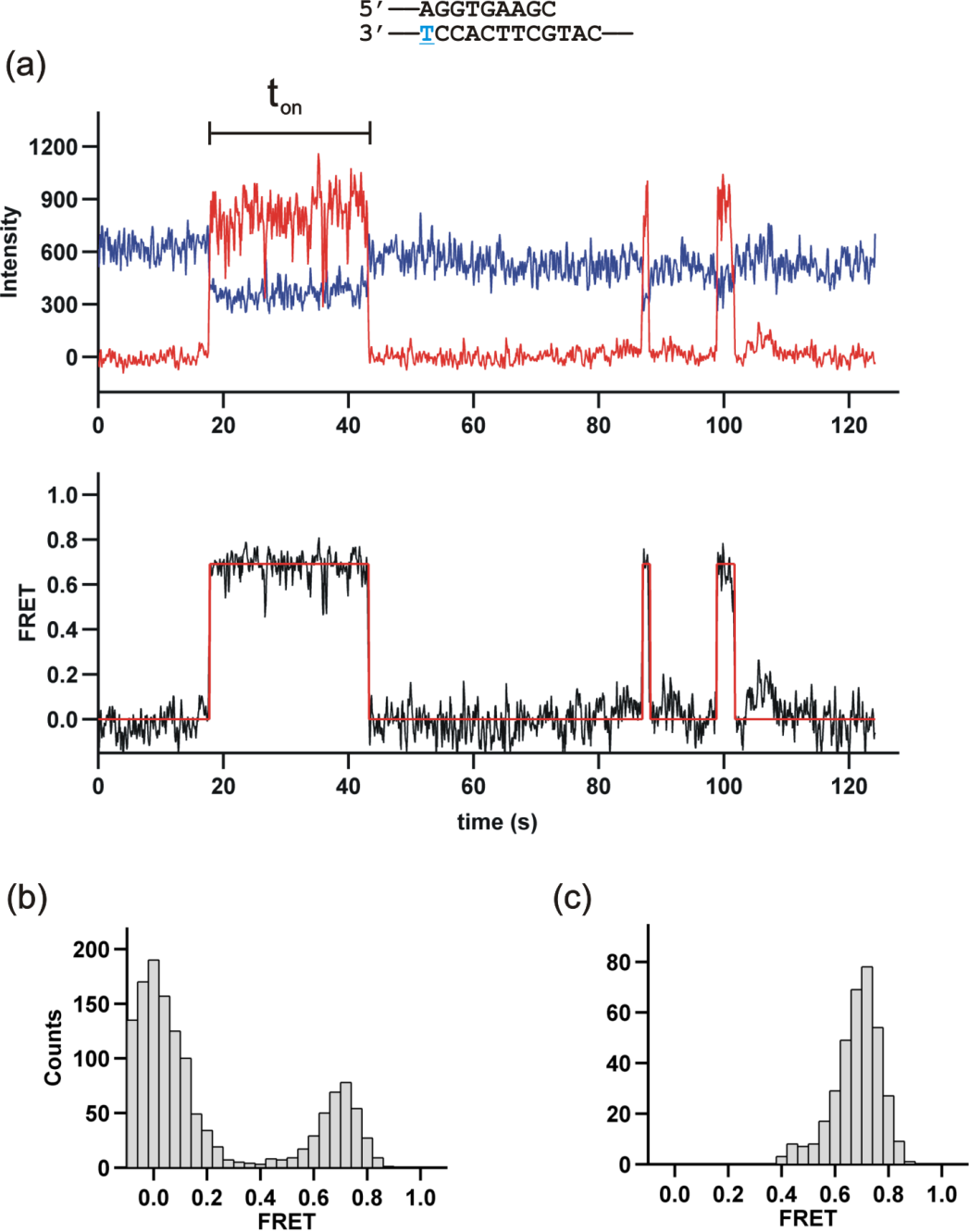


**Supplementary Figure S1.** Analysis of single-molecule FRET traces presented in this work. **(a)** Single-molecule FRET trace for Dpo4 binding the unmodified DNA duplex construct shown above. The top trace shows the real time intensities of donor (blue) and acceptor (red), while the bottom trace shows the FRET calculated as FRET = I_A_/(I_A_+I_D_). A Hidden Markov model (HMM) of the FRET trace is shown as a red line. All traces analyzed in this work were fit with a 2-state hidden Markov model. **(b)** Corresponding FRET histogram for the whole trace in (a). **(c)** Corresponding FRET histogram for the trace in (a) that excludes the points with a ~0 FRET determined by the HMM fit.

**Quantification of Gaussian peaks**

All FRET distributions were fit with one or two Gaussian peaks. The area under the curves was quantified and is shown in Tables S2-S5. FRET distributions with a single peak were excluded from this analysis.

**Supplementary Table S2**. Distributions and percentage Gaussian peak area for bimodal FRET distributions in Fig. 2.

|  | Unmodified | | AF | | AAF | |
| --- | --- | --- | --- | --- | --- | --- |
| (-)dCTP  FRET peak | 0.57 | 0.80 | 0.42 | 0.63 | 0.70 | 0.86 |
| % Area | 66.9 | 33.1 | 14.6 | 85.4 | 80.4 | 19.6 |
| (+)dCTP  FRET peak |  | | 0.55 | 0.70 | 0.63 | 0.86 |
| % Area |  | | 78.3 | 21.7 | 92.2 | 7.8 |

**Supplementary Table S3**. Distributions and percentage Gaussian peak area for bimodal FRET distributions in Fig. 3.

|  | Unmodified | | AF | | AAF | |
| --- | --- | --- | --- | --- | --- | --- |
| (-)dATP  FRET peak | 0.75 | 0.91 | 0.50 | 0.67 | 0.70 | 0.89 |
| % Area | 46.9 | 53.1 | 55.2 | 44.8 | 90 | 10 |
| (+)dATP  FRET peak |  | |  | | 0.61 | 0.87 |
| % Area |  | |  | | 94.2 | 5.8 |

**Supplementary Table S4**. Distributions and percentage Gaussian peak area for bimodal FRET distributions in Fig. 4.

|  | Unmodified | | AF | | AAF | |
| --- | --- | --- | --- | --- | --- | --- |
| FRET peak |  |  | 0.46 | 0.64 | 0.44 | 0.65 |
| % Area |  |  | 31.4 | 68.6 | 17.2 | 82.8 |

**Supplementary Table S5**. Distributions and percentage Gaussian peak area for bimodal FRET distributions in Fig. 5.

|  | Unmodified | | AF | | AAF | |
| --- | --- | --- | --- | --- | --- | --- |
| FRET peak |  |  |  |  | 0.47 | 0.66 |
| % Area |  |  |  |  | 30.7 | 69.3 |

**Interconversion between FRET states**

Bimodal FRET distributions may arise from interconversion between two states or static binding events to different FRET states. We analyzed all individual binding events with HMM and determined if they correspond to a single FRET state (Fig. S2a), or there is indeed interconversion between two different states (Fig. S2b). Results are summarized in Tables S6-9.


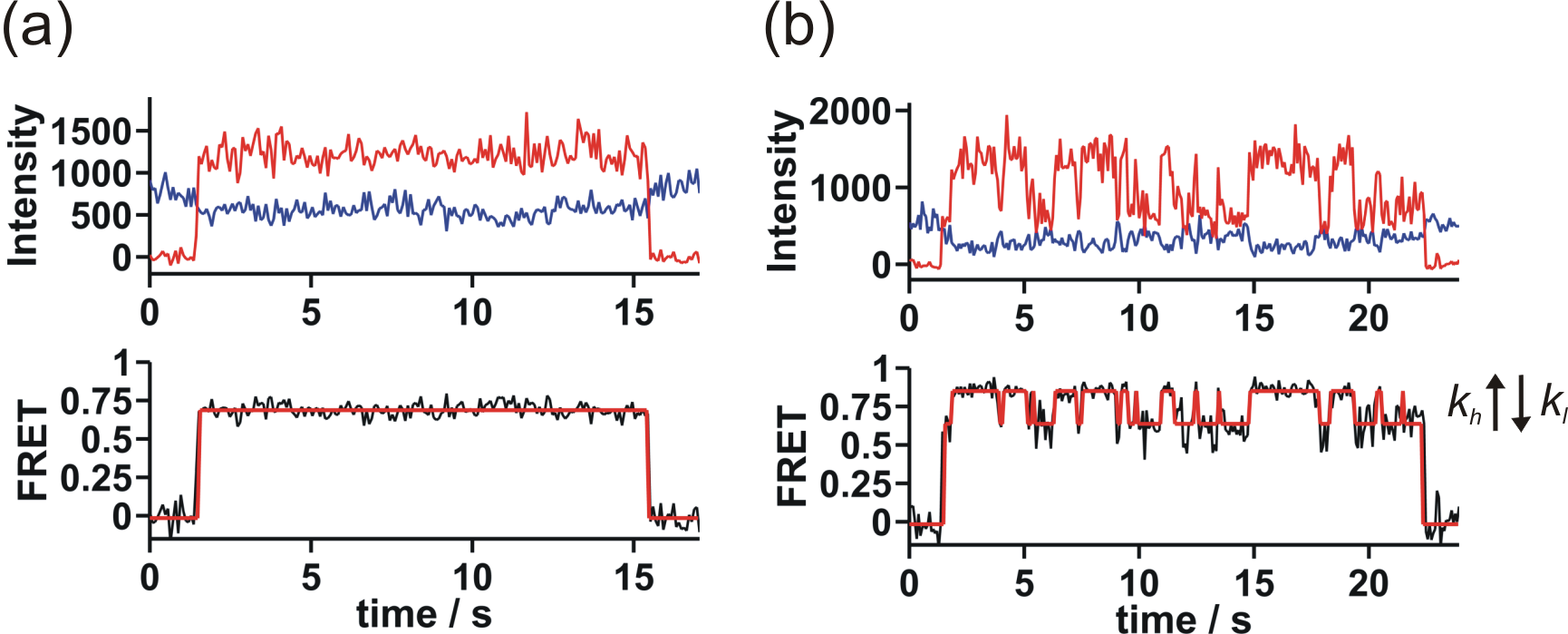


**Supplementary Figure S2.** Examples of static and dynamic binding events. **(a)** Static binding event where Dpo4/DNA complex samples a single FRET state. The HMM fit is shown as a red line. **(b)** Dynamic binding event with Dpo4/DNA complex sampling two FRET states. The HMM fit (red line) reconstructs the idealized trace and shows interconversion between FRET states centered at 0.6 and 0.8 respectively. Rate constant *k_h_* represents how fast the system goes from low to high FRET state and is obtained from the dwell-times in the low FRET state (0.6). *k_l_* corresponds to conversion from high to low FRET state and is obtained from the dwell-times in the high FRET state (0.8).

**Supplementary Table S6**. Percentage dynamic binding events for bimodal FRET distributions in Fig. 2. Interconversion rate constants *k_h_* and *k_l_* are also shown. The remaining percentages are static events.

|  | **(-)dCTP** | | | **(+)dCTP** | | |
| --- | --- | --- | --- | --- | --- | --- |
|  | Unm. | AF | AAF | Unm. | AF | AAF |
| % dynamic binding events | 76 | 54 | 43 |  | 26 | 40 |
| *k_h_* / s^-1^ | 4.78±0.12 | 5.12±0.13 | 1.8±0.3 |  | 4.9±0.4 | 1.2±0.2 |
| *k_l_* / s^-1^ | 5.8±0.3 | 1.27±0.07 | 1.4±0.2 |  | 0.69±0.12 | 2.7±0.4 |

**Supplementary Table S7**. Percentage dynamic binding events for bimodal FRET distributions in Fig. 3. Interconversion rate constants *k_h_* and *k_l_* are also shown.

|  | **(-)dATP** | | | **(+)dATP** | | |
| --- | --- | --- | --- | --- | --- | --- |
|  | Unm. | AF | AAF | Unm. | AF | AAF |
| % dynamic binding events | 64 | 44 | 66 |  |  | 42 |
| *k_h_* / s^-1^ | 5.6±0.2 | 3.4±0.3 | 3.9±0.3 |  |  | 2.1±0.2 |
| *k_l_* / s^-1^ | 5.5±0.3 | 7.4±0.4 | 2.4±0.3 |  |  | 4.6±0.3 |

**Supplementary Table S8**. Percentage dynamic binding events for bimodal FRET distributions in Fig. 4. Interconversion rate constants *k_h_* and *k_l_* are also shown.

|  | **(-)dNTP** | | |
| --- | --- | --- | --- |
|  | Unm. | AF | AAF |
| % dynamic binding events |  | 24 | 20 |
| *k_h_* / s^-1^ |  | 2.0±0.3 | 2.5±0.7 |
| *k_l_* / s^-1^ |  | 2.7±0.2 | 7.6±0.7 |

**Supplementary Table S9**. Percentage dynamic binding events for bimodal FRET distributions in Fig. 4. Interconversion rate constants *k_h_* and *k_l_* are also shown.

|  | **(-)dNTP** | | |
| --- | --- | --- | --- |
|  | Unm. | AF | AAF |
| % dynamic binding events |  |  | 19 |
| *k_h_* / s^-1^ |  |  | 1.1±0.7 |
| *k_l_* / s^-1^ |  |  | 2.3±1.2 |

**Quantification of gel bands**

Gel bands were quantified using ImageJ.

**Supplementary Table S10**. Band area percentage of the gel shown in Fig. 1b for the unmodified DNA reaction.

|  | Unmodified | | |
| --- | --- | --- | --- |
| Time / min | Primer | 33-mer | 34-mer |
| 2 | 11.8 | 61.8 | 26.4 |
| 5 | 10.0 | 26.0 | 64.0 |
| 10 | 14.4 | 4.8 | 80.8 |
| 20 | 16.9 | 0.0 | 83.1 |
| 45 | 8.9 | 0.0 | 91.1 |
| 90 | 10.9 | 0.0 | 89.1 |
| 150 | 8.0 | 0.0 | 92.0 |

**Supplementary Table S11**. Band area percentage of the gel shown in Fig. 1b for the AF-dG modified DNA reaction.

|  | AF | | | |
| --- | --- | --- | --- | --- |
| Time / min | 20-mer | 21-mer | 33-mer | 34-mer |
| 2 | 11.1 | 88.9 | 0.0 | 0.0 |
| 5 | 3.7 | 96.3 | 0.0 | 0.0 |
| 10 | 0.0 | 92.1 | 7.9 | 0.0 |
| 20 | 0.0 | 78.7 | 21.3 | 0.0 |
| 45 | 0.0 | 33.2 | 66.8 | 0.0 |
| 90 | 0.0 | 21.9 | 55.1 | 23.0 |
| 150 | 0.0 | 0.0 | 51.0 | 49.0 |

**Supplementary Table S12** Band area percentage of the gel shown in Fig. 1b for the AAF-dG modified DNA reaction.

|  | AAF | | | |
| --- | --- | --- | --- | --- |
| Time / min | 20-mer | 21-mer | 33-mer | 34-mer |
| 2 | 74.2 | 25.8 | 0.0 | 0.0 |
| 5 | 34.6 | 65.4 | 0.0 | 0.0 |
| 10 | 12.8 | 87.2 | 0.0 | 0.0 |
| 20 | 3.0 | 97.0 | 0.0 | 0.0 |
| 45 | 0.0 | 91.4 | 3.0 | 5.7 |
| 90 | 0.0 | 79.1 | 6.7 | 14.1 |
| 150 | 0.0 | 58.6 | 17.0 | 24.4 |

**Supplementary Table S13**. Percentage extension for the reactions shown in Fig. 3e.

| Template | Unmodified | | | | dG-AF | | | | dG-AAF | | | |
| --- | --- | --- | --- | --- | --- | --- | --- | --- | --- | --- | --- | --- |
| dNTP | A | C | G | T | A | C | G | T | A | C | G | T |
| % extension | 97.9 | 1.3 | 20.2 | 10.8 | 81.6 | 0.0 | 16.6 | 5.3 | 52.9 | 0.0 | 3.0 | 40.3 |

**Supplementary Table S14**. Percentage extension for the reactions shown in Fig. 4b.

| Template | Unmodified | | | | dG-AF | | | | dG-AAF | | | |
| --- | --- | --- | --- | --- | --- | --- | --- | --- | --- | --- | --- | --- |
| dNTP | A | C | G | T | A | C | G | T | A | C | G | T |
| % extension | 75.6 | 11.9 | 0.0 | 11.6 | 28.9 | 0.0 | 0.0 | 77.4 | 5.5 | 5.0 | 0.0 | 90.7 |

**Supplementary Table S15**. Percentage extension for the reactions shown in Fig. 5b.

| Template | Unmodified | | | | dG-AF | | | | dG-AAF | | | |
| --- | --- | --- | --- | --- | --- | --- | --- | --- | --- | --- | --- | --- |
| dNTP | A | C | G | T | A | C | G | T | A | C | G | T |
| % extension | 71.3 | 19.6 | 0.0 | 0.0 | 2.2 | 0.0 | 1.7 | 0.00 | 23.9 | 0.0 | 5.5 | 91.9 |

**
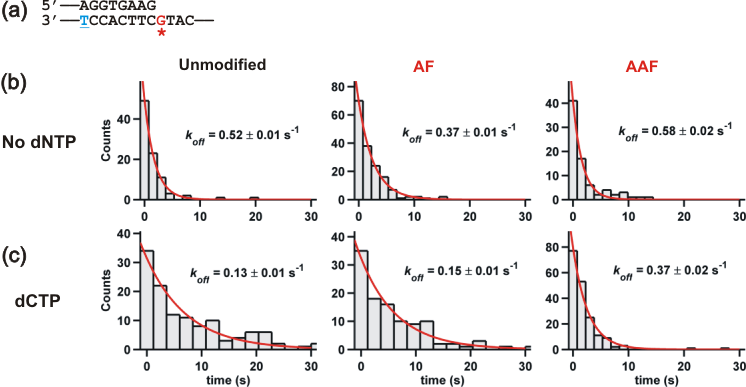
**

**Supplementary Figure S3.** Dpo4 ternary complex dissociates faster from an AAF-modified DNA construct compared to unmodified and AF-modified DNA. **(a)** DNA duplex sequence used in this set of experiments. **(b)** Dwell time histograms for Dpo4 binding the DNA show in (a) in the absence of nucleotides. **(c)** Dwell time histograms for Dpo4 binding the DNA show in (a) in the presence of 1 mM dCTP. A monoexponential fit (red line) yields the dissociation rate constant *k_off_* indicated in each graph.

**
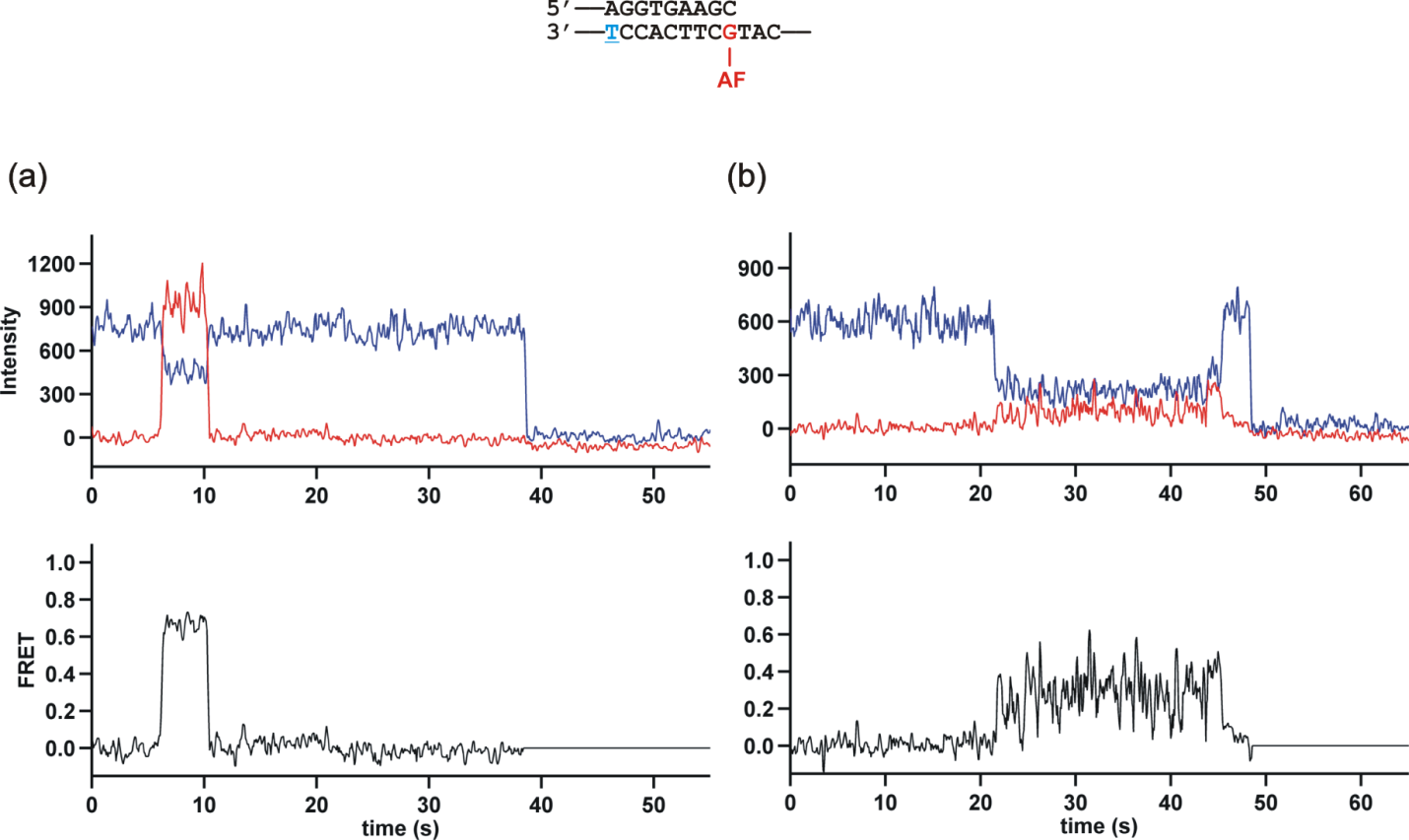
**

**Supplementary Figure S4.** Dpo4 binding to a 21mer-biotin primer/33mer-Cy3 template duplex in the absence of nucleotides yields two types of binding events. **(a)** Characteristic trace showing Dpo4 bound to DNA between time ~6 s and ~10 s. While Dpo4 is bound, Cy5 signal (red line) increases above the signal level of Cy3 (blue line) resulting in a FRET efficiency value of ~0.7. **(b)** In a second type of Dpo4 binding event, Cy3 fluorescence is decreased to a larger extent compared to (a), but the Cy5 intensity is barely increased above baseline level and does not reach above Cy3 signal. Dpo4 bound to DNA from time ~22 s to ~46 s yields a FRET efficiency value that oscillates between 0 and 0.5. All binding events corresponding to (b) type (approximately 25% for these experimental conditions) were discarded from the analysis and will be further investigated in future work.

**Supplementary Information References**

1. Lamichhane, R., Solem, A., Black, W. and Rueda, D. (2010) Single-molecule FRET of protein-nucleic acid and protein-protein complexes: surface passivation and immobilization. *Methods (San Diego, Calif.)*, **52**, 192-200.

2. Brenlla, A., Markiewicz, R.P., Rueda, D. and Romano, L.J. (2014) Nucleotide selection by the Y-family DNA polymerase Dpo4 involves template translocation and misalignment. *Nucleic acids research*, **42**, 2555-2563.

3. Zhao, R. and Rueda, D. (2009) RNA folding dynamics by single-molecule fluorescence resonance energy transfer. *Methods (San Diego, Calif.)*, **49**, 112-117.

4. McKinney, S.A., Joo, C. and Ha, T. (2006) Analysis of single-molecule FRET trajectories using hidden Markov modeling. *Biophysical journal*, **91**, 1941-1951.
